# Uncommon *Millettia pachycarpa* Benth poisoning: A case report

**DOI:** 10.1097/MD.0000000000038967

**Published:** 2024-07-26

**Authors:** Jiangchao Long, Yong Huang, Jun Guo

**Affiliations:** a Intensive Care Department, People’s Hospital of Dafang, Bijie, China; b Intensive Care Department, West China Hospital of Sichuan University, China.

**Keywords:** CRRT, diagnosis, *Millettia pachycarpa* Benth, poisoning, treatment

## Abstract

**Background:**

*Millettia pachycarpa* Benth, rich in rotenone, can disrupt the mitochondrial electron transport chain. Ingestion may cause respiratory and central nervous system depression, and in severe cases, lead to death. This is the first detailed clinical case report of *M. pachycarpa* Benth poisoning, aiming to help systematization of diagnosis and treatment.

**Patient concerns:**

An elderly male who lost consciousness for 3 hours after consuming the fruit of M. pachycarpa Benth. Arterial blood gas analysis indicated a significant decrease in pH, a sharp increase in lactate levels, and elevated CO2 partial pressure with normal O2 partial pressure.

**Diagnosis:**

The patient was diagnosed with food intoxication by M. pachycarpa Benth, concomitant with aspiration pneumonia and distributive shock.

**Interventions:**

The patient was given continuous renal replacement therapy (CRRT) and invasive mechanical ventilation.

**Outcomes:**

The patient was successfully discharged after 5 days of hospitalization. Follow-up after 2 weeks showed no significant discomfort.

**Conclusion:**

Isolated CO_2_ retention without hypoxemia, significantly reduced pH, and markedly elevated lactate levels strongly suggest poisoning by *M. pachycarpa* Benth. CRRT and invasive mechanical ventilation are beneficial for patients. Early implementation of CRRT to remove toxins and early initiation of assisted ventilation to improve respiratory failure are recommended upon suspicion of the disease.

## 1. Introduction

*Millettia pachycarpa* Benth, commonly known in Chinese as “kutanzi” (苦檀子), is a frequently encountered leguminous plant in China, predominantly found in the provinces of Guizhou, Sichuan, and Guangxi.^[1]^ It has been extensively utilized in traditional Chinese medicine as an anthelmintic and a topical agent for pain relief and bruising. The early symptoms of intoxication primarily involve the nervous system, ranging from mild dizziness, headache, numbness of the lips and limbs to severe respiratory depression, ataxia, and even shock and death. Despite ample food supply, sporadic cases of poisoning have been reported in China. Various toxic compounds, including rotenone and its analogs, can be isolated from the fruits and rhizomes of *M. pachycarpa* Benth.^[2]^ Rotenone is widely regarded as the main culprit of the toxic symptoms; as a lipophilic compound, it traverses biological membranes, including the blood–brain barrier, and directly affects cells by inhibiting the mitochondrial complex I of the electron transport chain.^[3]^ This action decreases adenosine triphosphate (ATP) formation, leading to cell death. Notably, this toxicity is nonselective, affecting all cells in an organism, with clinical features predominantly neurological due to the greater vulnerability of the central nervous system.^[4]^

This article reports a case of an elderly male who accidentally ingested the fruit of *M. pachycarpa* Benth, detailing the diagnostic and treatment process and reviewing clinical reasoning, in hopes of contributing to the future systematization of diagnosis and treatment.

## 2. Case report

The patient is a 70-year-old male with a history of coronary heart disease and persistent abnormalities on electrocardiogram: rapid ventricular rate atrial fibrillation with intraventricular conduction delay and ST-T changes. On March 11, 2024, he experienced weakness, nausea, vomiting, and diarrhea 3 hours after consuming wild fruits, followed by a gradual onset of consciousness disturbance and generalized seizures, without significant chills or fever. He was immediately brought to our hospital by his family.

### 2.1. Initial physical and auxiliary examinations upon hospital admission

Vital signs: Temperature 36.0°C, pulse 95 bpm, respiratory rate 18 bpm, blood pressure 85/56 mm Hg, oxygen saturation 98%.

Physical examination: The patient was unconscious and uncooperative. Pupils were equal, round, and measured approximately 2mm in diameter, with absent light reflex. There was mild cyanosis of the lips, the neck was supple, and the trachea was centrally positioned. There was a suspicion of a barrel-shaped chest; lung auscultation revealed clear breath sounds with scattered wet rales in the lower lobes bilaterally. The heart rate was 138 bpm with an irregular rhythm and variable intensity of the first heart sound; no valve murmurs were appreciable. The abdomen was flat, with no signs of guarding or rebound tenderness, and bowel sounds were noted at 4 times per minute. Limb muscle strength was not assessed, and muscle tone was normal. Physiological reflexes were intact, and pathological reflexes were negative bilaterally.

**Figure 1.**
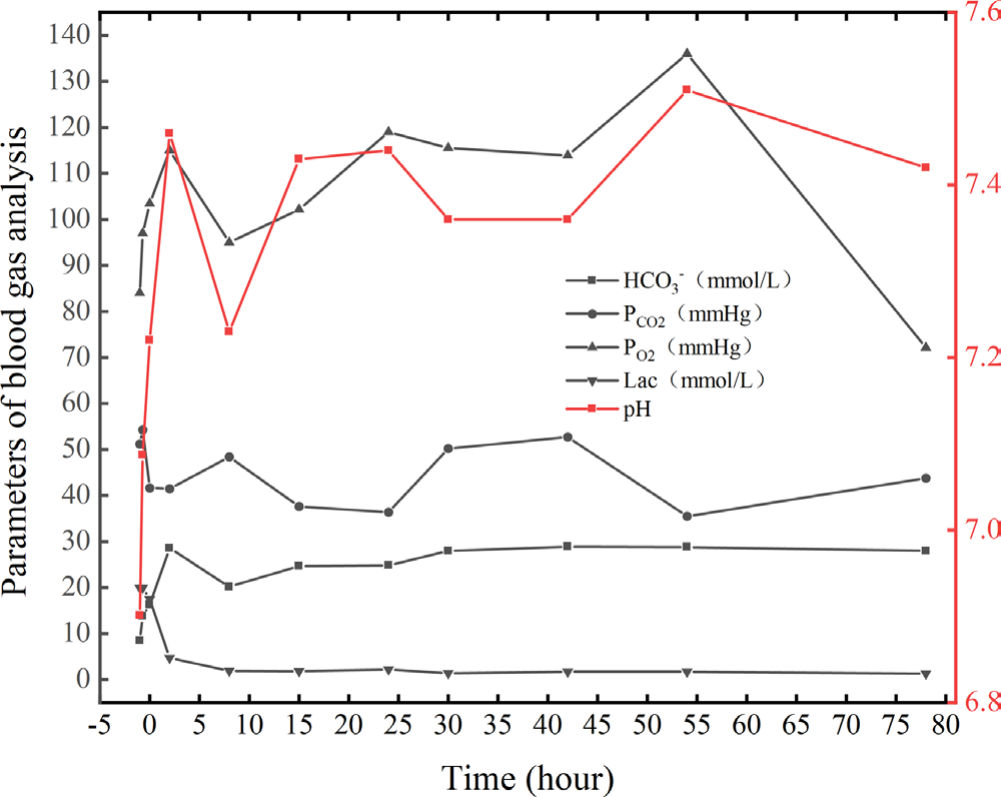
Arterial blood gas analysis results at different time.

Laboratory findings: Arterial blood gas analysis indicated pH 6.901, P_CO2_ 51.2 mm Hg, P_O2_ 84 mm Hg, HCO_3_^−^ 8.5 mmol/L, and base excess −23.5 mmol/L with lactate levels above the measurable range (>20 mmol/L); trends can be seen in Table 1 and Figure 1. Complete blood count showed white blood cell 22.98 × 10^9^/L, red blood cell 4.28 × 10^12^/L, platelets 402 × 10^9^/L, neutrophils 71.5%, and C-reactive protein (CRP) 14.05 mg/L. Coagulation profile, liver and renal functions, troponin, and atrial natriuretic peptide levels were within normal limits.

**Table 1:** Arterial blood gas analysis results at different time. (“0 h” represents admission to ICU, “−” represents before admission to ICU)

Time (h)	pH	HCO_3_^-^ (mmol/L)	P_CO2_ (mm Hg)	P_O2_ (mm Hg)	Lac (mmol/L)
−1	6.90	8.5	51.2	84	20
−0.6	7.087	13.8	54.3	97	20
0	7.22	16.3	41.6	103.4	17.5
2	7.46	28.6	41.5	115	4.7
8	7.23	20.2	48.4	95	1.9
15	7.43	24.7	37.6	102.2	1.8
24	7.44	24.8	36.4	119	2.2
30	7.36	28.0	50.2	115.53	1.4
42	7.36	28.9	52.7	113.9	1.6
54	7.51	28.8	35.5	136	1.7
78	7.42	28.0	43.75	72.15	1.3

**Figure 2.**
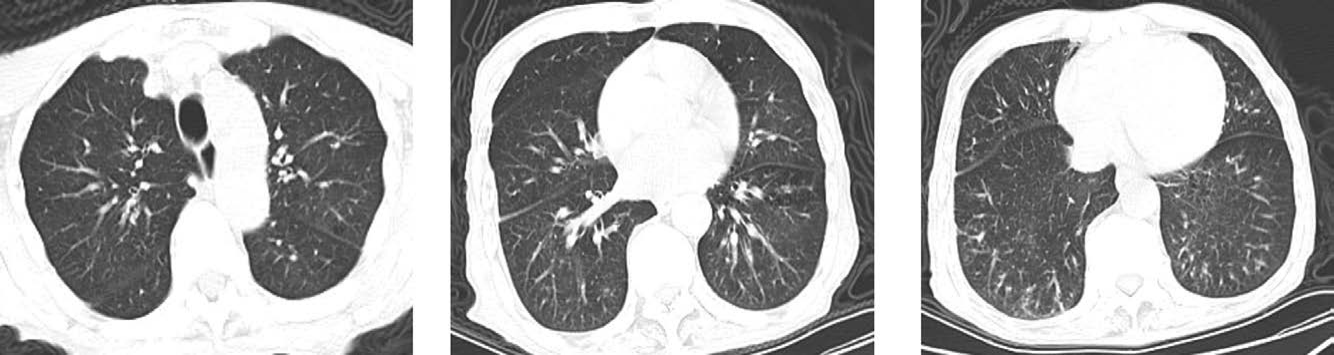
The patient’s admission chest CT scans. (left to right, representing upper, middle, and lower lung fields, reveal the presence of scattered inflammatory infiltrates).

Chest CT: Evidence of bilateral pulmonary inflammation suggestive of aspiration pneumonia, as depicted in Figure 2.

### 2.2. Diagnosis and management

#### 2.2.1. Pre-ICU assessment

**Figure 3.**
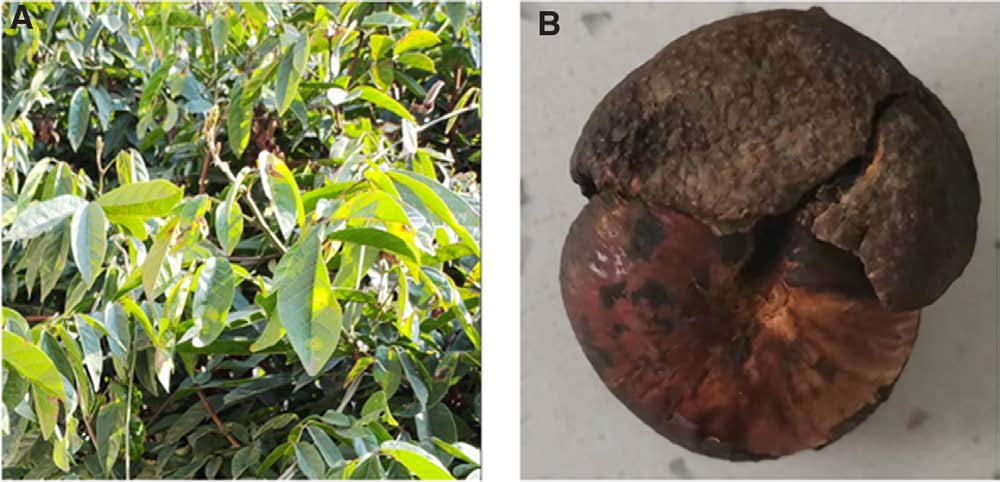
Leaves (A) and fruit (B) of *Millettia pachycarpa* Benth.

Prior to ICU admission, family members of the patient photographed the leaves and residual fruit of the plant (depicted in Figure 3), which were subsequently identified as the produce of *M. pachycarpa* Benth – a common regional flora. In the emergency department, utilizing the aforementioned visual evidence and clinical evaluations, the physicians established a diagnosis of food intoxication by *M. pachycarpa* Benth, concomitant with aspiration pneumonia and distributive shock. Initial interventions included the administration of sodium bicarbonate to correct metabolic acidosis among other supportive measures; however, the patient response remained suboptimal as indicated by a persistently low urine output (30 ml/h).

#### 2.2.2. ICU interventions

Within the initial hour of emergency department care, the patient necessitated transfer to the ICU, where arterial blood gas analyses were conducted, revealing pH 7.087, P_CO2_ 54.3 mm Hg, P_O2_ 97 mm Hg, H_CO3_^−^ 13.8 mmol/L, base excess −14.1 mmol/L, and lactate > 20 mmol/L. With no observable amelioration post presentation, endotracheal intubation was promptly performed. Mechanical ventilation was instituted (mode: AC/VC, Fi_O2_: 40%, respiratory rate: 18 bpm, tidal volume: 380 mL, PEEP: 7cm H_2_O), in conjunction with analgesia and sedation, managed by remifentanil and midazolam, achieving a RASS of −3 and a CPOT of 0. The multidisciplinary approach included the provision of tepid therapeutic hypothermia for cerebral protection, bicarbonate buffering for acid-base management, continuous infusion of noradrenaline (2.8 µg/kg/min) for hemodynamic stability, bedside renal support involving continuous veno-venous hemodiafiltration (mode: CVVHDF, anticoagulated: nafamostat mesylate, replacement fluids: 8000 ml, time: 5 hours), coupled with hemoperfusion (HA380 cartridge, time: 3 hours). The adjunctive therapeutic regimen entailed mannitol, omeprazole, inosine, cefotaxime, ambroxol, N-acetylcysteine, budesonide, and select traditional Chinese medicinal formulations.

Posttreatment, the patient’s systolic blood pressure consistently surpassed the 100mmHg mark independent of vasopressors. A singular session of continuous renal replacement therapy (CRRT) ordained no hepatic or renal sequelae while urine volume progressively improved to 50 mL/h. Subsequent to supportive measures including mechanical ventilation and hemoperfusion, an ongoing enhancement in blood gas parameters was observed (Table 1). Approximately 25 hours into the ICU stay, both the RASS and CPOT scores were normalized to 0. Patient consciousness was regained, accompanied by regularized muscular strength facilitating a strategic weaning from ventilatory support. Ventilator settings were adjusted (mode: CPAP, Fi_O2_: 40%, personality sensitivity: 8 cm H_2_O, PEEP: 6 cm H_2_O). 40 hours after admission to ICU, the patient satisfactorily cleared the preextubation assessment, which prefaced the successful discontinuation of mechanical ventilation (Table 1).

### 2.3. Convalescence and discharge

Postextubation, the patient transitioned to the geriatrics unit, evidencing steady recovery indicated by normal follow-up outcomes, culminating in discharge on March 17, 2024. The total duration of hospitalization from March 11th at 23:00 to discharge comprised 130 hours, inclusive of 1.5 hours in the emergency department, 81.5 hours in the ICU, and a subsequent 47 hours in geriatrics. Follow-up after 2 weeks showed no reports of discomfort or symptom reemergence.

## 3. Discussion

*M. pachycarpa* Benth commonly thrives within evergreen broad-leaved forests on slopes below an elevation of 2000 m, where unsuspecting individuals might consume the plant unknowingly due to unfamiliarity with its appearance or ignorance of its toxicity, leading to poisoning. Clinical symptoms of this poisoning are nonspecific, primarily because the principal toxicological action of rotenone, the active component, is the inhibition of Iron–Sulfur (Fe–S) clusters, resulting in mitochondrial dysfunction.^[5]^ This attack is nonselective; however, due to the vulnerability of neural cells, neurotoxicity may manifest initially. Rotenone has a molecular weight of 394.42 Da, is lipophilic, and is found in a variety of plants; it is widely regarded as closely associated with the onset of Parkinson disease.^[6]^

Diagnosis of poisoning by this plant is challenging and fraught with potential misidentification. This difficulty is not only attributable to the nonspecific nature of the symptoms but also to frequent confusion between *M. pachycarpa* Benth and *Spatholobus suberectus* Dunn by patients or healthcare professionals, as both plants exhibit similarity in appearance when dried and are utilized as traditional Chinese medicinal herbs. In this case report, definitive identification was achieved after consultation with professional cultivators of medicinal herbs. In addition, we found errors in both Chinese and English research articles that misidentified the plant as *S. suberectus* Dunn and used the *M. pachycarpa* Benth folkloric name “jixueteng(鸡血藤)” for it. While both plants belong to the Fabaceae family, they exhibit marked differences in leaf and fruit morphology; notably, the fruit of *M. pachycarpa* Benth is large and plump, resembling an egg, whereas the fruit of *S. suberectus* Dunn is narrow and elongated, akin to a sickle. In traditional Chinese medicine, their pharmacological applications are vastly different, with the former primarily used as an insecticide,^[2]^ while the latter is known to promote blood circulation.^[7]^

From laboratory examinations, distinctive characteristics of such poisonings can be discerned, notably an aberrant arterial blood gas profile: isolated carbon dioxide retention without hypoxemia, significantly reduced pH, and marked elevation in lactate levels. Clinically, respiratory failure is typically categorized into hypoxemic (type I) or hypercapnic (type II),^[8]^ the latter often being a consequence of ventilatory rather than diffusive limitations. Despite not presenting with traditional hypoxemia, this patient was diagnosed with type II respiratory failure due to mitochondrial dysfunction. Inadequate ventilation compounded by ineffective oxygen utilization resulted in seemingly normal Pa_O2_ levels, while Pa_CO2_ was elevated. The reduced pH corroborates our hypothesis, as metabolic acidosis typically prompts hyperventilation and a subsequent decrease in Pa_CO2_^[9]^; however, this compensatory response was not observed in the patient. Lactate production is determined by 3 factors: production of pyruvate, its utilization, and lactate clearance.^[10]^ Elevated lactate postexercise stems from increased anaerobic glycolysis, while chronic renal failure elevates lactate due to reduced clearance. Given that neither of these 2 factors influenced this patient, the marked deficiency in pyruvate utilization strongly suggests poisoning by *M. pachycarpa* Benth or similar toxins.

Distributive shock is diagnosed based on hypotension and severe acidosis after other shock etiologies have been excluded. Elevated lactate in shock patients is largely attributable to inadequate perfusion leading to disrupted pyruvate utilization.^[11,12]^ Acidosis further exacerbates this deficiency, where excess protons cause abnormal electromotive differences across smooth muscle cell membranes, resulting in excessive vasodilation of the microvasculature and further compromised effective circulating volume.^[13,14]^ This perpetuates a positive feedback loop that requires swift intervention to interrupt. In circulatory shock management, addressing the underlying cause is paramount,^[15]^ which is why invasive mechanical ventilation and CRRT were initiated upon the patient’s ICU admission. Adequate ventilation alleviates both metabolic (reduction in lactate) and respiratory acidosis (reduction in CO_2_ levels), while timely CRRT can adjust for acid-base and electrolyte imbalances and facilitate toxin removal via hemoperfusion. While the HA380 perfusion device, with a filtration range of 10 to 60 kDa,^[16]^ does not directly remove rotenone due to its smaller molecular weight, it can eliminate the toxin by adsorbing the proteins rotenone binds to. The HA380 is also capable of adsorbing a variety of inflammatory mediators and is widely used in the treatment of septic shock.^[17]^ Macrophage migration inhibitory factor, a secretory homotrimer with a key role in pro-inflammatory cytokine production and a molecular weight of 12.5 kDa,^[18]^ can be directly adsorbed by the device, rendering hemoperfusion a necessary treatment. No adverse and unanticipated events were found during hospitalization or after discharge. However, the duration and frequency of CRRT should be individualized; this patient underwent a single session because there was continuous improvement in pH, increased urine output, and no observed risk of latent hepatic or renal dysfunction. Economic factors were also considered, as the patient’s family was unwilling to expend additional funds on nonessential CRRT treatments.

The limitations of this study are that we have made theoretical extrapolations from the clinical characteristics of only 1 patient, and more cases are needed for validation; and, although the therapeutic efficacy of CRRT as well as hemoperfusion is promising, its true clinical value may need to be confirmed by large-scale multicenter prospective trials.

## 4. Conclusion

*M. pachycarpa* Benth is uncommon in regions beyond Asia, with a conspicuous absence of detailed clinical case reports or research addressing its toxicological impact. Analysis of our case revealed distinctive hallmarks of *M. pachycarpa* Benth toxicity: isolated hypercapnia without hypoxemia, a notable plummet in pH, and a substantial rise in lactate concentrations. Clinically, the employment of CRRT coupled with invasive mechanical ventilation has been shown to afford therapeutic benefit to afflicted patients. This positive outcome has been observed even in geriatric patients with preexisting coronary artery disease, with a comprehensive treatment duration averaging around 5 days, and subsequent follow-ups revealing no significant discomfort. Consequently, healthcare practitioners should promptly resort to CRRT for detoxification and initiate assisted ventilation at the earliest suspicion of such pathology to ameliorate respiratory failure.

## Acknowledgments

We are grateful for the useful comments and suggestions of anonymous referees.

## Author contributions

**Data curation:** Jiangchao Long, Yong Huang.

**Formal analysis:** Jiangchao Long, Yong Huang.

**Investigation:** Jiangchao Long.

**Resources:** Jiangchao Long, Yong Huang.

**Software:** Jiangchao Long, Yong Huang.

**Validation:** Jiangchao Long.

**Writing – original draft:** Jiangchao Long, Yong Huang.

**Methodology:** Yong Huang, Jun Guo.

**Conceptualization:** Jun Guo.

**Supervision:** Jun Guo.

**Writing – review & editing:** Jun Guo.

## References

[1] Tu Y Wu C Kang Y Li Q Zhu C Li Y. Bioactivity-guided identification of flavonoids with cholinesterase and β-amyloid peptide aggregation inhibitory effects from the seeds of Millettia pachycarpa. Bioorg Med Chem Lett. 2019;29:1194–8. 30910460 10.1016/j.bmcl.2019.03.024

[2] Suthiphasilp V Rujanapun N Kumboonma P. Antidiabetic and Cytotoxic Activities of Rotenoids and Isoflavonoids Isolated from *Millettia pachycarpa* Benth. ACS Omega. 2022;7:24511–21. 35874225 10.1021/acsomega.2c02163 PMC9301698

[3] Ibarra-Gutiérrez MT Serrano-García N Orozco-Ibarra M. Rotenone-induced model of Parkinson’s disease: beyond mitochondrial complex I inhibition. Mol Neurobiol. 2023;60:1929–48. 36593435 10.1007/s12035-022-03193-8

[4] Sanfeliu C Bartra C Suñol C Rodríguez-Farré E. New insights in animal models of neurotoxicity-induced neurodegeneration. Front Neurosci. 2023;17:1248727. 38260026 10.3389/fnins.2023.1248727 PMC10800989

[5] Read AD Bentley RE Archer SL Dunham-Snary KJ. Mitochondrial iron-sulfur clusters: structure, function, and an emerging role in vascular biology. Redox Biol. 2021;47:102164. 34656823 10.1016/j.redox.2021.102164 PMC8577454

[6] Bisbal M Sanchez M. Neurotoxicity of the pesticide rotenone on neuronal polarization: a mechanistic approach. Neural Regen Res. 2019;14:762–6. 30688258 10.4103/1673-5374.249847 PMC6375050

[7] Huang X Fei Q Yu S. A comprehensive review: botany, phytochemistry, traditional uses, pharmacology, and toxicology of Spatholobus suberectus vine stems. J Ethnopharmacol. 2023;312:116500. 37062528 10.1016/j.jep.2023.116500

[8] Lagina M Valley TS. Diagnosis and management of acute respiratory failure. Crit Care Clin. 2024;40:235–53. 38432694 10.1016/j.ccc.2024.01.002 PMC10910131

[9] Arena A Miller E. Respiratory acid-base disorders. Emerg Med Clin North Am. 2023;41:863–75. 37758429 10.1016/j.emc.2023.06.009

[10] Müller J Radej J Horak J. Lactate: the fallacy of oversimplification. Biomedicines. 2023;11:3192. 38137413 10.3390/biomedicines11123192 PMC10741081

[11] De Backer D Ricottilli F Ospina-Tascón GA. Septic shock: a microcirculation disease. Curr Opin Anaesthesiol. 2021;34:85–91. 33577205 10.1097/ACO.0000000000000957

[12] De Backer D Arias Ortiz J Levy B. The medical treatment of cardiogenic shock: cardiovascular drugs. Curr Opin Crit Care. 2021;27:426–32. 33797431 10.1097/MCC.0000000000000822

[13] Boedtkjer E Ara T. Strengthening the basics: acids and bases influence vascular structure and function, tissue perfusion, blood pressure, and human cardiovascular disease. Pflugers Arch. 2024;476:623–37. 38383822 10.1007/s00424-024-02926-z

[14] Klöckner U Isenberg G. Calcium channel current of vascular smooth muscle cells: extracellular protons modulate gating and single channel conductance. J Gen Physiol. 1994;103:665–78. 8057083 10.1085/jgp.103.4.665 PMC2216859

[15] Soussi S Dos Santos C Jentzer JC. Distinct host-response signatures in circulatory shock: a narrative review. Intensive Care Med Exp. 2023;11:50. 37592121 10.1186/s40635-023-00531-5 PMC10435428

[16] Pomarè Montin D Ankawi G Lorenzin A Neri M Caprara C Ronco C. Biocompatibility and cytotoxic evaluation of new sorbent cartridges for blood hemoperfusion. Blood Purif. 2018;46:187–95. 29886501 10.1159/000489921

[17] Hellman T Uusalo P Järvisalo MJ. Renal replacement techniques in septic shock. Int J Mol Sci . 2021;22:10238. 34638575 10.3390/ijms221910238 PMC8508758

[18] Yang J Ji D Zhu YQ. Hemoperfusion with HA380 in acute type A aortic dissection patients undergoing aortic arch operation (HPAO): a randomized, controlled, double-blind clinical trial. Trials. 2020;21:954. 33228727 10.1186/s13063-020-04858-2 PMC7684885

